# Complete gallbladder torsion diagnosed with sequential computed tomography scans: a case report

**DOI:** 10.1186/1752-1947-6-289

**Published:** 2012-09-11

**Authors:** Takahiro Koyanagi, Kaoru Sato

**Affiliations:** 1 Department of Obstetrics and Gynecology, Jichi Medical University, 3311-1, Yakushiji, Shimotsuke-shi, Tochigi Pref., 329-0431, Japan; 2 Department of Surgery, Katta General Hospital, 36, Shimookihara, Fukuokakuramoto, Shiroishi-shi, Miyagi Pref., 398-0231, Japan

**Keywords:** Acute abdomen, Cholecystectomy, Diagnostic imaging, Gallbladder torsion

## Abstract

**Introduction:**

Torsion of the gallbladder is an extremely rare cause of acute abdomen, which commonly affects thin elderly women. A prompt surgical approach is necessary to avoid fatal complications associated with gangrene and perforation of the gallbladder. However, it is difficult to make a preoperative diagnosis using ordinary imaging modalities.

**Case presentation:**

An 84-year-old Japanese woman was admitted to our hospital due to left lower abdominal pain. Her pain shifted suddenly to the right upper abdomen a half day after admission. Although her enlarged and wall-thickened gallbladder had been already seen at admission, it rotated approximately 180 degrees and deviated to the midline of her abdomen on the second computed tomography scan, which helped us to make a correct diagnosis of gallbladder torsion. The patient underwent an emergency operation (detorsion and cholecystectomy) and recovered without any complications. The gallbladder had necrosis due to torsion.

**Conclusion:**

Sequential diagnostic imaging might be helpful to make a preoperative diagnosis of gallbladder torsion when the gallbladder is enlarged and wall thickened but the patient does not present with typical clinical symptoms.

## Introduction

Torsion of the gallbladder is an extremely rare condition that was first reported in 1898 by Wendel [1]. It has been reported to occur more commonly in thin elderly women and the incidence appears to be increasing, possibly related to an increasingly ageing population [2,3]. Clinical symptoms and signs of gallbladder torsion include severe right upper abdominal pain and vomiting with sudden onset, palpable abdominal mass, and absence of jaundice and fever [4]. The results of laboratory investigations including liver function tests and biliary enzymes are usually within normal limits [5]. Although prompt surgery is necessary to avoid high mortality associated with gangrene and perforation of the gallbladder, it is difficult to make a preoperative diagnosis of gallbladder torsion.

We report here a case of torsion of the gallbladder in an elderly woman without typical clinical symptoms, in which sequential computed tomography (CT) scans helped us to make a correct diagnosis preoperatively.

## Case presentation

**Figure 1:**
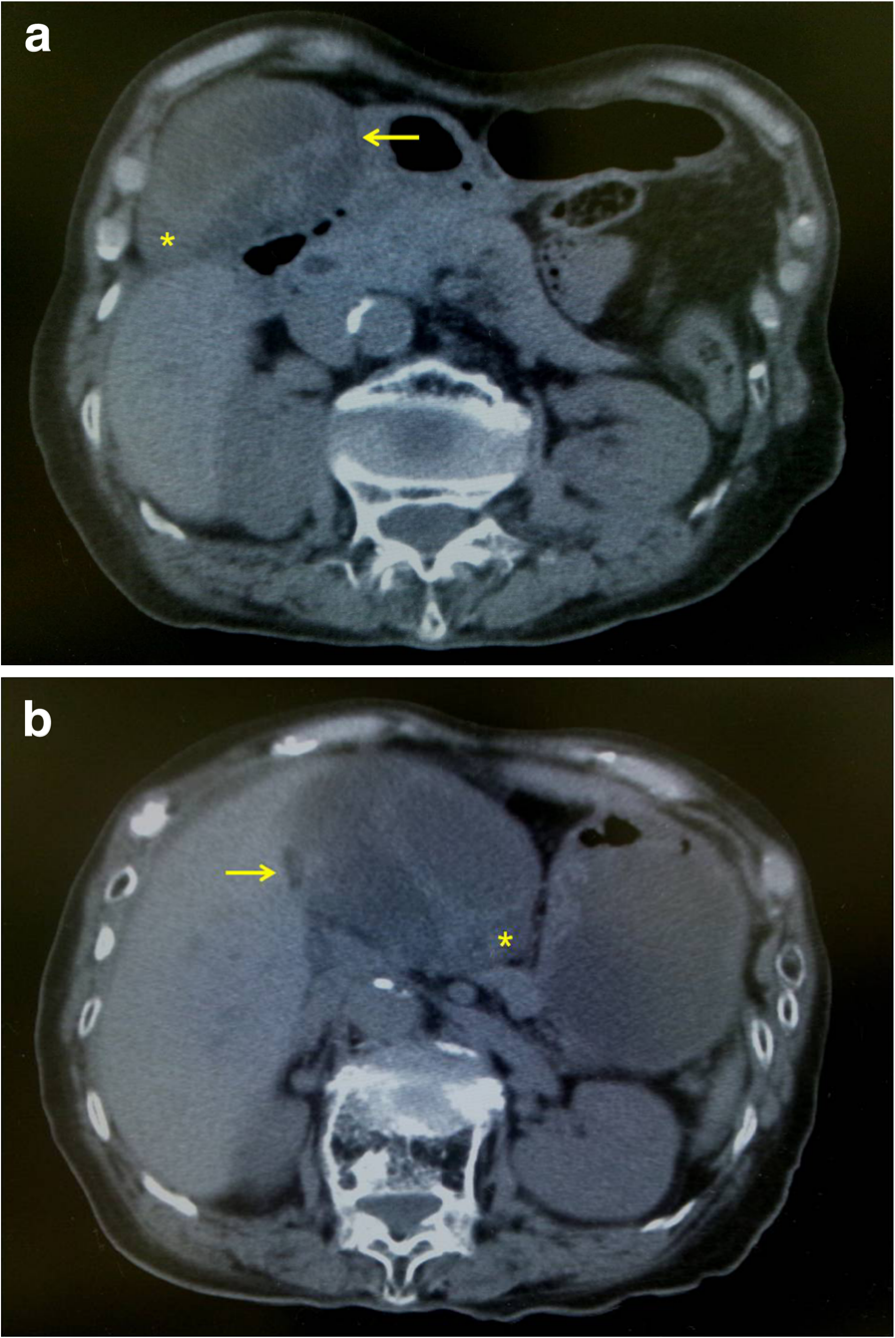
**(a) Computed tomography scan of the patient’s abdomen at admission.** The gallbladder was enlarged and wall thickened without gallstones, and it was located in its normal anatomical fossa. The arrow shows the cystic duct before rotation. The asterisk shows the gallbladder fundus before rotation. (**b**) Computed tomography scan of the patient’s abdomen a half day after admission. The gallbladder had rotated approximately 180 degrees and deviated to the midline of the abdomen. It became even more distended and wall thickened. The cystic duct was located on the right side of the gallbladder (arrow). The asterisk shows the gallbladder fundus after rotation.

**Figure 2:**
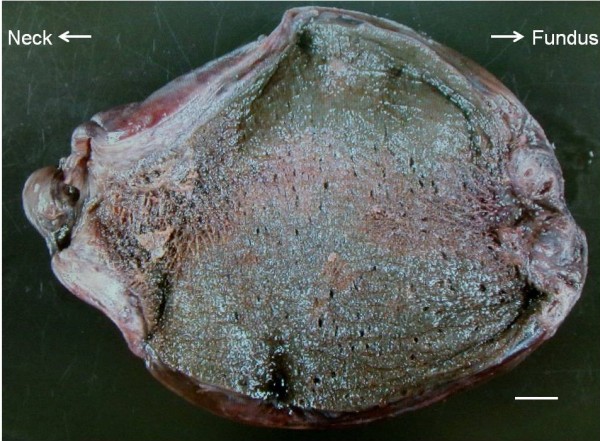
**Gross finding of the patient’s resected gallbladder.** The gallbladder appeared to be hugely enlarged (fist-sized), wall thickened, and necrotic. Scale bar, 1cm.

An 84-year-old woman was admitted to our hospital with complaints of left lower abdominal pain and nausea without fever. Her past surgical history included a hysterectomy and a femoral neck fracture repair. She also suffered from hypertension and hyperlipidemia. Her body mass index was 18.2kg/m^2^ and her vital signs were all within normal limits. Physical examination demonstrated tenderness in the left lower abdomen without Blumberg’s sign and with no demonstration of Murphy’s sign. Laboratory data were all within normal limits including inflammatory response, liver function tests, and biliary enzymes. A CT scan of her abdomen at admission demonstrated an enlarged and wall-thickened gallbladder without gallstone, prominent small bowel gas, and mild intestinal dilatation and wall thickening (Figure 1a). The findings of ultrasonography were the same as those of the CT scan. Therefore, we diagnosed her condition as acute enterocolitis or subileus and began a starvation and transfusion cure. After a half day, she suddenly presented with severe right upper abdominal pain and vomiting. Conservative treatment, including a painkiller and antiemetic, was not effective so another abdominal CT scan was carried out. The second CT scan of her abdomen demonstrated a more distended and wall-thickened gallbladder that had rotated approximately 180 degrees and deviated to the midline of her abdomen compared with the CT images at admission (Figure 1b). The cystic duct was located on the right side of the gallbladder. Taking these into account, we considered that her gallbladder was free hanging and a diagnosis of gallbladder torsion was clinically assumed. Therefore, the patient underwent an emergency laparotomy. During the operation we observed a gallbladder torsion (rotation more than 180 degrees) leading to necrosis and we performed detorsion and cholecystectomy. The gallbladder was free hanging and adhesion to adjacent organs was so mild that operative procedures were easy to perform. Pathological examination revealed necrotic change, compatible with an acute bleeding infarction of the gallbladder due to torsion (Figure 2). There was no evidence of malignancy. The patient recovered without surgical complications but suffered from postoperative delirium. She was discharged about two weeks after surgery.

## Discussion

There are two types of gallbladder torsion [6]: one is incomplete torsion (rotation less than 180 degrees) with gradual onset, and the other is complete torsion (rotation more than 180 degrees) with acute onset. When this patient was admitted to our hospital, her chief complaint was left lower abdominal pain and no right upper abdominal pain was complained of; her enlarged and wall-thickened gallbladder was seen on the CT scan. We therefore diagnosed her condition as acute enterocolitis or subileus and chose conservative treatment. Evaluating retrospectively, the gallbladder torsion was considered incomplete at the time of admission. After a half day, she suddenly presented with a severe right upper abdominal pain. The second CT scan showed that the gallbladder had rotated approximately 180 degrees and had deviated to the midline of her abdomen. Taking these findings into account, at this time her gallbladder was thought to be free hanging and a diagnosis of gallbladder torsion was clinically assumed. Actually, complete torsion was observed during an emergency operation. Although it is unclear why she presented with left lower abdominal pain at first, it is possible that enterocolitis or subileus occurred due to the intestinal inflammation spreading from the torsed gallbladder.

The decisive findings of gallbladder torsion are described in the literature as left-sided enlarged gallbladder, thickened gallbladder wall without enhancement effect, and a cystic duct located on the right side of the gallbladder [7,8]. However, it is quite rare for clinicians to make a correct preoperative diagnosis of torsion of the gallbladder based on ordinary radiographic findings. Therefore, as in the present case, chronological diagnostic imaging including CT scans might be helpful when a patient presents with gradual clinical symptoms or atypical symptoms such as lower abdominal pain with an enlarged and wall-thickened gallbladder. In addition, it has been increasingly reported that novel imaging modalities, such as multi-detector row CT, magnetic resonance imaging (MRI), and magnetic resonance cholangiopancreatography also help preoperative diagnosis [9].

As for the treatment for torsion of the gallbladder, prompt detorsion and cholecystectomy are required to avoid potentially fatal sequelae of gangrene and perforation of the gallbladder [3]. We chose open cholecystectomy in the present case because we could not completely exclude the possibility of bile duct malignancy (painless gallbladder swelling and wall thickening in an elderly patient). However, the effectiveness of laparoscopic cholecystectomy has also been reported [10]. Because adhesion between the gallbladder and the adjacent organs is usually mild, which may facilitate gallbladder torsion, it is relatively easy to dissect the gallbladder from the liver bed and to cut the cystic duct and cystic artery. Therefore, laparoscopic procedures are recommended for the treatment of gallbladder torsion.

## Conclusions

Torsion of the gallbladder is a rare cause of acute abdomen and it is difficult to diagnose preoperatively. It is important to take this disease into consideration in the differential diagnosis of acute abdomen in all elderly patients. Moreover, sequential diagnostic imaging including CT scans might be helpful when the gallbladder is enlarged and wall thickened but the patient does not present with typical clinical symptoms.

## Consent

Written informed consent was obtained from the patient for publication of this case report and any accompanying images. A copy of the written consent is available for review by the Editor-in-Chief of this journal.

## Competing interests

The authors declare that they have no competing interests.

## Authors’ contributions

TK and KS diagnosed, investigated and managed the patient. Also, TK and KS determined the medical significance and wrote the manuscript. All authors read and approved the final manuscript.

## References

[B1] Wendel AV VI. A case of floating gall-bladder and kidney complicated by cholelithiasis, with perforation of the gall-bladder Ann Surg 1898 27 199 202 17860545 PMC1426674

[B2] Nakao A Matsuda T Funabiki S Mori T Koguchi K Iwado T Matsuda K Takakura N Isozaki H Tanaka N Gallbladder torsion: case report and review of 245 cases reported in the Japanese literature J Hepatobiliary Pancreat Surg 1999 6 418 421 10.1007/s005340050143 10664294

[B3] Ijaz S Sritharan K Russell N Dar M Bhatti T Ormiston M Torsion of the gallbladder: a case report J Med Case Reports 2008 2 237 10.1186/1752-1947-2-237 PMC2503992 18652648

[B4] Haines FX Kane JT Acute torsion of the gallbladder Ann Surg 1948 128 253 256 10.1097/00000658-194808000-00008 17859195 PMC1513732

[B5] Shaikh AA Charles A Domingo S Schaub G Gallbladder volvulus: report of two original cases and review of the literature Am Surg 2005 71 87 89 15757065

[B6] Carter R Thompson RJ Brennan LP Hinshaw DB Volvulus of the gallbladder Surg Gynecol Obstet 1963 116 105 108 14018964

[B7] Merine D Meziane M Fishman EK CT diagnosis of gallbladder torsion J Comput Assist Tomogr 1987 11 712 713 10.1097/00004728-198707000-00032 3597901

[B8] Aibe H Honda H Kuroiwa T Yoshimitsu K Irie H Shinozaki K Mizumoto K Nishiyama K Yamagata N Masuda K Gallbladder torsion: case report Abdom Imaging 2002 27 51 53 10.1007/s00261-001-0050-7 11740608

[B9] Matsuhashi N Satake S Yawata K Asakawa E Mizoguchi T Kanematsu M Kondo H Yasuda I Nonaka K Tanaka C Misao A Ogura S Volvulus of the gallbladder diagnosed by ultrasonography, computed tomography, coronal magnetic resonance imaging and magnetic resonance cholangiopancreatography World J Gastroenterol 2006 12 4599 4601 16874883 10.3748/wjg.v12.i28.4599 PMC4125658

[B10] Kim SY Moore JT Volvulus of the gallbladder: laparoscopic detorsion and removal Surg Endosc 2003 17 1849 1852 14959731 10.1007/s00464-002-4521-x

